# The Influence of the *CES1* Genotype on the Pharmacokinetics of Enalapril in Patients with Arterial Hypertension

**DOI:** 10.3390/jpm12040580

**Published:** 2022-04-05

**Authors:** Anna Ikonnikova, Tatiana Rodina, Artem Dmitriev, Evgeniy Melnikov, Ruslan Kazakov, Tatiana Nasedkina

**Affiliations:** 1 Engelhardt Institute of Molecular Biology, Russian Academy of Sciences, 119991 Moscow, Russia; tanased06@rambler.ru; 2 Federal State Budgetary Institution “Scientific Centre for Expert Evaluation of Medicinal Products” of the Ministry of Health of the Russian Federation, 127051 Moscow, Russia; semina_tatiana@mail.ru (T.R.); dmitriev-doc@mail.ru (A.D.); rustic100@rambler.ru (R.K.); 3 Institute of Pharmacy of I. M. Sechenov First MSMU of the Ministry of Health of the Russian Federation (Sechenov University), 119435 Moscow, Russia; melnikov_e_s@staff.sechenov.ru

**Keywords:** *CES1*, carboxylesterase 1, enalapril, SNP, pharmacogenetics, cardiology, hypertension

## Abstract

The angiotensin-converting enzyme inhibitor enalapril is hydrolysed to an active metabolite, enalaprilat, in the liver via carboxylesterase 1 (CES1). Previous studies show that variant rs71647871 in the *CES1* gene affects the pharmacokinetics of enalapril on liver samples as well as healthy volunteers. This study included 286 Caucasian patients with arterial hypertension who received enalapril. The concentrations of enalapril and enalaprilat were determined before subsequent intake of the drug and 4 h after it with high-performance liquid chromatography (HPLC) and mass spectrometric detection. The study included genetic markers as follows: rs2244613, rs71647871 (c.428G>A, p.G143E) and three SNPs indicating the presence of a subtype *CES1A1c* (rs12149368, rs111604615 and rs201577108). Mean peak and trough enalaprilat concentrations, adjusted by clinical variables, were significantly lower in *CES1* rs2244613 heterozygotes (by 16.6% and 19.6%) and in CC homozygotes (by 32.7% and 41.4%) vs. the AA genotype. In *CES1A1c* homozygotes, adjusted mean enalaprilat concentrations were 75% lower vs. heterozygotes and wild-type (WT) homozygotes. Pharmacogenetic markers of the *CES1* gene may be a promising predictor for individualisation when prescribing enalapril.

## 1. Introduction

The angiotensin-converting enzyme inhibitor (ACEI) enalapril is a widely used antihypertensive drug prescribed to treat arterial hypertension and chronic heart failure. Although new antihypertensive drugs have been developed and are entering clinical practice, enalapril remains the critical drug for the therapy of chronic heart failure [1].

Enalapril is an ethyl ester prodrug, hydrolysed to an active metabolite enalaprilat in the liver by the serine esterase carboxylesterase 1 (CES1) [2]. Enalaprilat has a strong hypotensive effect, due to its ability to inhibit the angiotensin-converting enzyme (ACE). The severity and duration of the hypotensive effect of enalapril is largely determined by the rate of its hydrolysis to enalaprilat, varying significantly in patients. Interindividual differences in the metabolism of enalapril are largely associated with activity of the CES1 enzyme. Various single nucleotide polymorphisms (SNPs) in the *CES1* gene on its activity were studied against substrates of this enzyme: ACE inhibitors [3,4,5,6], clopidogrel [7,8,9], dabigatran [10,11,12,13,14,15], methylphenidate [16,17], oseltamivir [18,19,20], capecitabine [21,22], isoniazid [23], etc.

The most studied polymorphism in the *CES1* gene is c.428G>A (rs71647871), which leads to the amino acid change Gly143Glu and to loss of enzyme function. It was shown that in carriers of allele A, activity of the enzyme is reduced [3,4,7,8,9,16,18,19]. These are mainly heterozygous carriers, as the frequency of the minor allele is low.

Another promising pharmacogenetic marker in the *CES1* gene is the intronic variant 1168-33C>A (rs2244613). Various studies show decreased enzyme function and associated clinical outcomes in carriers of the C allele [11,13,21,24,25].

A subtype *CES1A1c* was described as a variant with reduced function [26], with its promoter region containing exon 1 with adjoining sequences derived from a pseudogene *CES1P1*; this is determined by several SNPs in high-linkage disequilibrium [23]. However, further results were seen as inconsistent [3,6].

As for enalapril, studies of the pharmacokinetic impact of *CES1* gene variants were carried out in vitro on liver samples [3] or on healthy volunteers [4,5,6]. Our study was carried out on a cohort of patients with arterial hypertension who received enalapril to treat it, and who underwent therapeutic drug monitoring (TDM). We studied the effect of three genetic markers in the *CES1* gene (rs2244613 and rs71647871 and the variant *CES1A1c*) on peak and trough plasma enalaprilat concentration.

## 2. Materials and Methods

### 2.1. Patients

We included 286 patients (100 men and 186 women, aged 41 to 90 years) with grades 1–3 of arterial hypertension (Table 1) who received enalapril in doses of 2.5 to 20 mg twice a day. The prescribed enalapril dose was based on the grade of arterial hypertension and on the treatment regimen at admission. Enalapril was taken as an antihypertensive monotherapy in 71 patients, and some were taking other antihypertensive drugs such as hydrochlorothiazine, indapamide, nifedipine and amlodipine. Co-medications taken by patients are listed in Appendix A. All patients were Caucasians in the Moscow region. Renal function was assessed by the level of the glomerular filtration rate (GFR), calculated with the CKD-EPI calculator [27]. Patients with GFR < 15 mL/min (chronic kidney disease stage 5) were not included in the study. Enalapril dose titration was performed, based on TDM and daily monitoring of blood pressure. For all patients, the target blood pressure level (<130/80 mm Hg) was achieved with treatment.

This study was approved by the Institutional Review Board of the Federal State Budgetary Institution “Scientific Centre for Expert Evaluation of Medicinal Products” of the Ministry of Health of the Russian Federation (protocol № 2018/04 and date of approval 17 January 2018). All participants provided written informed consent. The study was performed in accordance with the World Medical Association Declaration of Helsinki.

### 2.2. Pharmacokinetic Study Design and Determination of Drug Concentrations

On day three, while taking enalapril, each patient underwent TDM. Blood samples for determination of the plasma concentrations of enalapril and enalaprilat were collected before the following intake of the drug (i.e., 12 h after the previous intake) and 4 h after it. For enalaprilat, these values correspond to peak and trough concentrations, respectively.

The analysis used the triple quadrupole liquid chromatograph-mass spectrometer Nexera LCMS-8040 (QQQ) (Shimadzu, Kyoto, Japan). One-step protein precipitation with 50% trifluoroacetic acid solution was used for blood serum sample preparation. Calibration samples were prepared by spiking blank human serum with enalapril maleate (Sigma-Aldrich St. Louis, MO, USA) and enalaprilat (Sigma-Aldrich) mixed working solutions. Promethazine hydrochloride (Sigma-Aldrich, USA) was used as an internal standard. Chromatographic separation was carried out on a Synergy Polar RP, 50 × 2 mm, 4 μm, 80 Å column (Phenomenex, Torrance, CA, USA) with a C18 universal guard, 4 × 3.0 mm (Phenomenex) at a temperature of 40 °C. The mobile phase consisted of eluent A (1%, *v*/*v* formic acid in deionized water) and eluent B (1%, *v*/*v* formic acid in acetonitrile). The volume of the injected sample was 2 μL. Separation was carried out in the gradient mode by solvent composition and by flow rate within 3 min (Supplementary Methods, Tables S3 and S4). The retention time for enalapril was about 1.04 min; for enalaprilat, about 0.94 min; and for promethazine, about 1.20 min. Analytes detection was performed using positive electrospray ionization (ESI) and multiple reaction monitoring (MRM). MRM transitions were 377.20 > 234.20 m/z for enalapril, 349.30 > 117.10 m/z for enalaprilat and 285.10 > 86.10 m/z for promethazine. The analytical range for both enalapril and enalaprilat was 5 ng/mL to 250 ng/mL. This method development and validation have been described in detail [28]. Some patients underwent several measurements of pharmacokinetic parameters for dose selection, as data on the last measurement were included in the genetic study. For one patient, there was no measurement value before taking the drug (for technical reasons).

### 2.3. DNA Extraction

Genomic DNA was extracted from blood collected in EDTA-containing tubes using the QIamp DNA Mini kit (Qiagen, Hilden, Germany) or the LumiPure genomic DNA Blood and Buccal Kit (Lumiprobe RUS Ltd., Moscow, Russia) according to the manufacturer’s guidelines. The DNA concentration and quality were estimated with a NanoDrop 1000 spectrophotometer (Thermo Fisher Scientific, Waltham, MA, USA).

### 2.4. CES1 Genotyping

We developed a low-density biochip to determine *CES1* genotypes for these markers: rs2244613 (c.1171−33C>A), rs71647871 (c.428G>A, p.G143E) and 3 SNPs that indicate the presence of a subtype *CES1A1c* (c.−2C>G rs12149368, c.11G>C—rs111604615, c.16T>C—rs201577108) [29]. The rs71647871 and rs2244613 regions were amplified using genomic DNA, and long-range/nested PCR detected *CES1A1c*. Biochip technology was described earlier [30]; 2′-deoxyuridine 5′-triphosphate (dUTP) derivatives containing the Cy7 cyanine dye were used as a fluorophore [31]. Analysis procedure, primers, oligonucleotide probes and analysis are in the Supplementary Methods, Tables S1 and S2, Figures S1 and S2. Genotyping validation was performed by Sanger sequencing. Sequencing primers and conditions are also in the Supplementary Methods. The *CES1* rs2244613 genotypes were validated with the kit «GenTest CES1» (Nomotekh, Moscow, Russia).

### 2.5. Statistical Analysis

Multivariable linear regression analysis with stepwise variable selection could evaluate the effect of selected genetic markers (*CES1A1c*, rs2244613 and rs71647871) on peak and trough enalaprilat concentrations. Non-genetic variables in the model initially included single dose, age, gender, GFR, weight and body mass index (BMI). Enalaprilat concentration and a single dose were log-transformed to fit the linear regression assumptions (natural logarithm, ln). Null values (19 samples) were removed in the analysis of trough concentration, as they interfered with the correctness of the model; in addition, from studied variables, the only difference between groups of null and non-null values turned out to be a single dose. Figures show log-transformed enalaprilat concentration (for trough concentrations, as values are presented as ln(x + 1) due to null values), and tables show those untransformed.

The online service SNPStats (https://www.snpstats.net/start.htm?, the last accessed date 1 April 2022) [32] was used to evaluate differences in adjusted enalaprilat concentration means (adjusted for significant variables as a corresponding linear regression model) between different genotypes. The comparison of baseline characteristics in groups with different genotypes was completed with the Kruskal–Wallis test and the Chi-Square test. The effect of genotype on the similarity of enalaprilat concentrations with the therapeutic range was performed with a two-tailed Fisher’s exact test. Statistical analysis and visualisation were performed in R software (packages “ggplot2,” “gvlma”) and SNPStats [32]. Differences were considered statistically significant when the p-value was below 0.05.

## 3. Results

Two hundred eighty-six samples were genotyped for selected SNPs. All samples with identified minor alleles, such as A rs71647871, were reamplified by region from the promoter to intron 5 of *CES1* (12.5 kb) as the template, then sequenced to determine heterozygous or homozygous carriage [33]. The genotyping results for all markers corresponded to the Hardy–Weinberg equilibrium (Table 2). No differences in baseline characteristics (age, gender, GFR, weight, BMI, single dose) were found between genotypes (Supplementary Results, Table S5).

Dose, age, gender and one genetic marker *CES1* rs2244613 were statistically significant predictors in the final linear regression model for peak enalaprilat concentration (Table 3). *CES1A1c* in the homozygous carriage was “at the level of the trend towards significance.” Mean peak enalaprilat concentrations adjusted by dose, age and gender were 16.6% lower in heterozygotes and 32.7% lower in CC vs. the AA genotype, or 16.4% lower per C allele (*p* = 0.027 for codominant model, *p* = 0.0072 for log-additive model).

For trough concentration, significant predictors in the linear regression model were dose, age and two genetic markers: *CES1* rs2244613 and *CES1A1c* (Table 4). Adjusted mean trough enalaprilat concentrations were 19.6% lower in heterozygotes and 41.4% lower in CC homozygotes vs. the AA genotype of *CES1* rs2244613 or 20% lower per C allele (*p* = 0.022 for the codominant model, *p* = 0.0056 for log-additive model). In *CES1A1c* homozygotes, adjusted mean enalaprilat concentrations were 75% lower vs. heterozygotes and wild-type (WT) homozygotes (*p* = 0.043).

**Figure 1:**
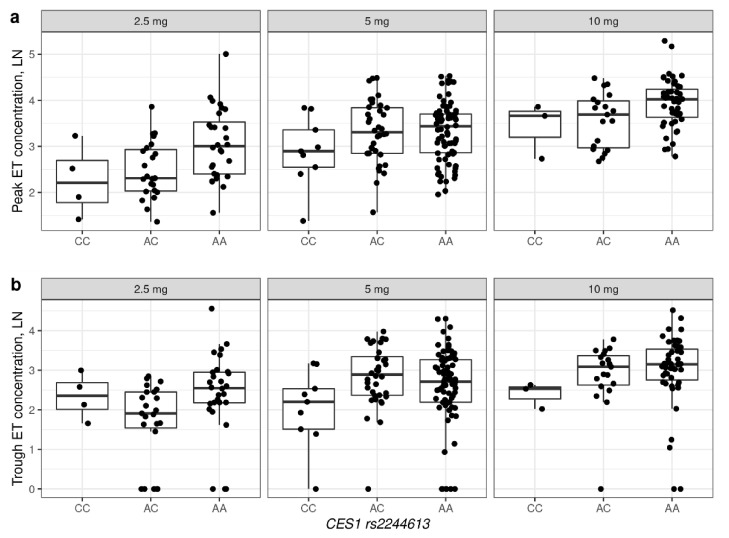
Peak (**a**) and trough (**b**) concentrations of enalaprilat, based on *CES1* rs2244613 genotypes in patients receiving different doses of enalapril. The figure shows concentrations for which there was more than one observation for each genotype. ET—enalaprilat. Ln—natural logarithm.

**Figure 2:**
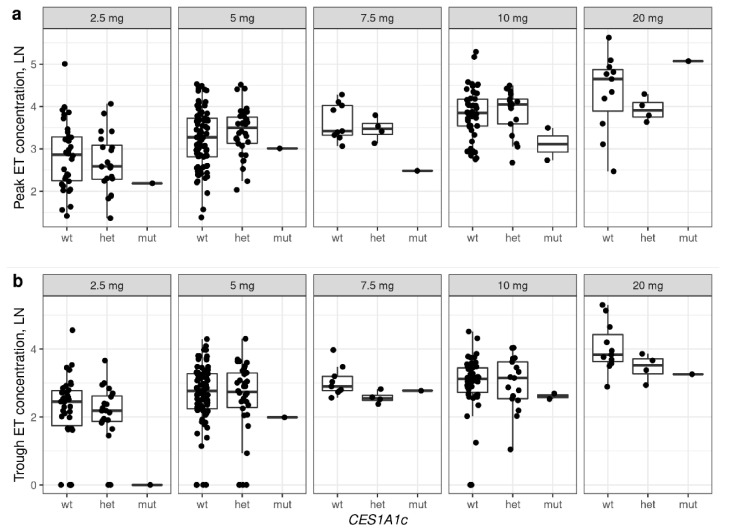
Peak (**a**) and trough (**b**) concentrations of enalaprilat, based on *CES1A1c* genotypes in patients receiving different doses of enalapril. The figure shows concentrations for which there was at least one observation for each genotype. ET—enalaprilat. WT—wild-type homozygous, wild-type heterozygous (WT/*CES1A1c*), mut—*CES1A1c* homozygous (*CES1A1c*/*CES1A1c*). Ln—natural logarithm.

The association of plasma enalaprilat concentrations for rs2244613 and *CES1A1c* are illustrated in Figure 1 and Figure 2, respectively. No association was found between enalaprilat concentrations and rs71647871. Descriptive statistics for concentrations of enalaprilat, based on genotypes and single doses, are presented in the (Supplementary Results Table S6).

**Figure 3:**
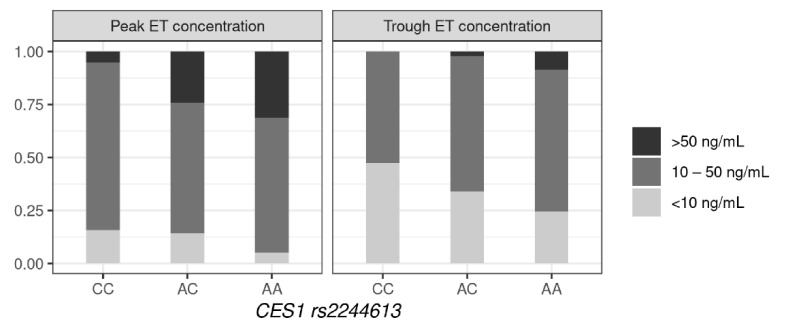
Similarity of enalaprilat concentrations in the therapeutic range, based on the *CES1* rs2244613 genotype.

As is known, the therapeutic range for enalaprilat is from 10 to 50 ng/mL [34]. The distribution of patients with different genotypes depends on whether the concentration of enalaprilat is in the therapeutic range, as shown in Figure 3 and the (Supplementary Results Table S7). In patients with the CC and AC genotypes, the concentration of enalaprilat was lower than the therapeutic range more often than the AA genotype—both for peak and trough concentration (OR = 3.22, 95%CI = 1.35–7.69, *p* = 0.0063 and OR = 1.78, 95%CI = 1.06–3.03, *p* = 0.029). Exceeding the therapeutic range was typical for carriers of the A allele—as statistically significant differences were observed in the AA and AC genotypes compared to the CC for peak concentration (OR = 7.29, 95%CI = 0.96–55.6, *p* = 0.03), as well as in the AA genotype compared to the CC and AC for trough concentration (OR = 5.06, 95%CI = 1.13–22.59, *p* = 0.02).

Based on TDM, as well as clinical effect and adverse reactions, the dose of enalapril was adjusted: increased, decreased or the drug was canceled. As many patients were taking several antihypertensive drugs, this study was unable to compare how clinical decisions depended on genotype.

## 4. Discussion

To our knowledge, our study is the first to investigate the effect of *CES1* genotypes on the pharmacokinetics of enalapril in patients with arterial hypertension receiving enalapril for antihypertensive therapy. Accordingly, our study cohort was a heterogeneous group of patients differing in many ways, such as weight, age, kidney function and prescribed drug dose. Regression analysis assessed the influence of genetic factors, with clinical parameters.

In our research, rs2244613 was the most significant marker in the *CES1* gene. Both peak and trough plasma concentrations of enalaprilat were reduced in carriers of the C allele, with the decrease more pronounced in homozygotes than heterozygotes. This variant had been investigated for its effect on plasma angiotensin II/angiotensin I ratio in patients with congestive heart failure who received ACEIs, which showed no significant effect [35], but its effect on ACEI pharmacokinetics has not been studied. However, studies on other drugs that are CES1 substrates showed that rs2244613 can have a significant effect on enzyme activity [11,13,21,24,25]. The mechanism of this effect is unknown, as this variant is intronic and does not affect protein sequence. Since allele C frequency is high, and the decrease in enzyme function and its impact on clinical parameters is pronounced in heterozygotes and homozygotes, we consider it to be a promising pharmacogenetic marker.

We showed that in homozygotes for the *CES1A1c* variant, the trough concentration of enalaprilat was significantly reduced. This individuals also had reduced peak concentrations, but this was not statistically significant. It is worth noting that the p-values were given without correction for multiple comparison. Given that we examined three genetic markers, after adjusting for multiple comparisons, *CES1A1c* becomes non-significant, whereas rs2244613 remains statistically significant. As per our data, the effect of *CES1A1c* is significantly manifested in homozygotes, and their frequency is quite low (~2%). Another limitation is that detection of *CES1A1c* requires nested PCR, as it is not convenient for testing, and its significance must be high to be meaningful for implementation in clinical practice. Yet, data on *CES1A1c* are contradictory. The *CES1A1c* variant was investigated in two studies on the pharmacokinetics of enalapril and did not show a statistically significant effect on the drug’s metabolism [3,6].

In our study, the loss-of-function variant rs71647871 (c.428G>A) had no effect on enalaprilat plasma concentrations. In previous studies on liver samples in vitro [3] and in healthy volunteers [4,5], it was found that allele A reduces the metabolism of enalapril to enalaprilat, though one study did not show this association [6]. In our cohort, allele A was found in 7 patients (2.5%). Perhaps other factors had a greater influence and levelled its effect.

For clinical factors, the most statistically significant effect on peak and trough plasma concentrations of enalaprilat was exerted by dosage and age. In the study cohort, age was expected to be inversely correlated with GFR (Pearson correlation coefficient = −0.48, *p*-value < 2.2 × 10^−16^); therefore, an increase in enalaprilat plasma concentrations with age may be associated with a decrease in GFR. For peak concentration, gender was a significant predictor—it was lower in men than women. It was previously reported that CES1 activity in the liver of women may be higher than in men [14,18].

This study has some limitations. Firstly, it is unable to assess the effect of genotype on the severity of the antihypertensive effect of enalapril and adverse drug reactions, since most patients took several antihypertensive drugs. Further, in addition to the studied genetic markers, other markers in the *CES1* gene and other genes involved in the metabolism of enalapril could contribute, as well as non-genetic factors such as drug–drug interactions and comorbidities. The subject requires further research.

In conclusion, enalapril is a well-known drug that has long been used to treat arterial hypertension. We found that with empirical selection of the enalapril dosage, the concentration of the active metabolite may not reach the therapeutic range or, to the contrary, is higher. Markers in the *CES1* gene seem to have a significant impact on the pharmacokinetics of enalapril and may help in a rational choice of therapy.

## Figures and Tables

**Table 1:** Baseline characteristics of patients.

Characteristics	All Patients (*n* = 286)
Gender, *n* (%)	
women	186 (65%)
men	100 (35%)
Age, years (mean ± sd)	67.22 ± 10.13
Weight, kg (mean ± sd)	84.6 ± 18.95
BMI (mean ± sd)	30.89 ± 6.15
GFR, mL/min (mean ± sd)	72.09 ± 20.08
Grade of arterial hypertension, *n* (%)	
1	10 (3.5%)
2	46 (16.1%)
3	230 (80.4%)
CAD, *n* (%)	140 (49%)
CHF, *n* (%)	110 (38.5%)
AF, *n* (%)	97 (33.9%)
DM type 2, *n* (%)	93 (32.5%)
Dislipidemia, *n* (%)	128 (44.8%)
Single enalapril dose, *n* (%):	
2.5 mg	55 (19.2%)
5 mg	121 (42.3%)
7.5 mg	14 (4.9%)
10 mg	69 (24.1%)
12.5 mg	2 (0.7%)
15 mg	9 (3.1%)
20 mg	16 (5.6%)

AF—atrial fibrillation, BMI—body mass index, CAD—coronary artery disease, CHF—chronic heart failure, DM—diabetes mellitus, GFR—glomerular filtration rate, sd—standard deviation.

**Table 2:** *CES1* variants frequencies in studied patients.

Genopype	*n*, %	HWE χ2 *p*-Value
*CES1A1c*		
wt	197 (69%)	0.42
wt/*CES1A1c*	83 (29%)	
*CES1A1c/CES1A1c*	6 (2%)	
*CES1* rs71647871		
GG	279 (97.5%)	0.83
GA	7 (2.5%)	
AA	0 (0%)	
*CES1* rs2244613		
AA	176 (61.6%)	0.13
AC	91 (31.8%)	
CC	19 (6.6%)	

HWE—Hardy–Weinberg equilibrium.

**Table 3:** Linear regression analysis on peak enalaprilat concentration.

	Coefficient	SE	*p*-Value	
Intercept	1.413	0.292	2.10 × 10^−6^	***
Age	0.013	0.004	0.000756	***
Single dose, ln	0.733	0.064	<2 × 10^−16^	***
Gender_male	−0.192	0.08	0.016837	*
*CES1* rs2244613_AC	−0.181	0.081	0.026514	*
*CES1* rs2244613_CC	−0.435	0.153	0.004704	**
wt/*CES1A1c*	−0.003	0.083	0.97459	
*CES1A1c/CES1A1c*	−0.492	0.262	0.061048	.
Adjusted R-squared: 0.3679, *p*-value: <2.2 × 10^−16^				

ln—natural logarithm, SE—standard error. For genetic predictors, wild-type homozygotes were the reference. Significance codes (*p*-value): “***”—<0.001, “**”—<0.01, “*”—<0.05, “.”—<0.1.

**Table 4:** Linear regression analysis on trough enalaprilat concentration.

	Coefficient	SE	*p*-Value	
Intercept	0.384	0.303	0.20680	
Age	0.023	0.004	1.17 × 10^−8^	***
Single dose, ln	0.556	0.069	2.34 × 10^−14^	***
*CES1* rs2244613_AC	−0.12	0.087	0.16724	
*CES1* rs2244613_CC	−0.49	0.161	0.00263	**
wt/*CES1A1c*	−0.111	0.088	0.20873	
*CES1A1c/CES1A1c*	−0.605	0.294	0.04088	*
Adjusted R-squared: 0.2957, *p*-value: <2.2 × 10^−16^				

ln—natural logarithm, SE—standard error. For genetic predictors, wild-type homozygotes were the reference. Significance codes (*p*-value): “***”—<0.001, “**”—<0.01, “*”—<0.05.

## Data Availability

The data presented in this study are available on request from the corresponding author.

## Supplementary Materials

The following supporting information can be downloaded at: https://www.mdpi.com/article/10.3390/jpm12040580/s1, S1: Supplementary methods; S2: Supplementary results. Table S1. Mobile phase composition in chromatographic separation; Table S2. Flow rates in chromatographic separation; Table S3 Oligonucleotide probes immobilized on a biochip; Table S4. Primers for amplification of rs71647871 and rs2244613 for biochip analysis; Table S5. Baseline characteristics of patients between genotypes *CES1*; Table S6. Peak and trough enalaprilat concentrations based on *CES1* genotypes in patients receiving different doses of enalapril; Table S7. Distribution of patients according to correspondence of the therapeutic range of en-alaprilat, depending on the *CES1* rs2244613 genotype; Figure S1. Biochip scheme; Figure S2. An example of analysis on a biochip.

## Appendix A. List of Co-Medications Taken by Patients, *n* (%)

Calcium channel blockers—120 (42%)
Loop diuretics—42 (14.7%)
Potassium-sparing diuretics—45 (15.7%)
Thiazides and thiazide-like diuretics—94 (32.9%)
Losartan—18 (6.3%)
Digoxin—20 (7%)
β-blockers—232 (81.1%)
Statins—217 (75.9%)
Aspirin—119 (41.6%)
Warfarin—28 (9.8%)
Rivaroxaban—45 (15.7%)
Dabigatran—12 (4.2%)
Metformin—54 (18.9%)
Other glucose-lowering drugs—38 (13.3%)
Proton-pump inhibitors (PPIs)—57 (19.9%)
