# Supplementary material for: The Influence of the CES1 Genotype on the Pharmacokinetics of Enalapril in Patients with Arterial Hypertension
Source: J Pers Med. 2022 Apr 5;12(4):580. doi: 10.3390/jpm12040580 (PMC9028383; doi:10.3390/jpm12040580)
Supplement: Supplementary file 1 [file jpm-12-00580-s001.zip › jpm-1641398-supplementary.pdf]

SUPPLEMENTARY MATERIALS

S1. Supplementary methods……………………………………………………….. Pages 2-6
S2. Supplementary results ………………………………………………………… Pages 6-8

1. Supplementary Methods

1.1. Chromatographic separation

Table S1. Mobile phase composition in chromatographic separation

Time, min	B, % v/v
0.00 → 0.20	5
0.20 → 0.21	5 → 37
0.21 → 0.90	37 → 39
0.90 → 0.91	39 → 47
0.91 → 1.45	47
1.45 → 1.86	47 → 100
1.86 → 2.10	100
2.10 → 2.20	100 → 5
2.20 → 3.00	5

Table S2. Flow rates in chromatographic separation

Time, min	Flow rate, mL/min
0.00 → 0.20	0.4
0.20 → 0.21	0.40 → 0.60
0.21 → 0.75	0.60
0.75 → 0.76	0.60 → 0.90
0.76 → 2.25	0.90
2.25 → 2.26	0.90 → 0.40
2.26 → 3.00	0.40

1.2. Biochip scheme and oligonucleotide probes

Figure S1. Biochip scheme

**rs12149368**<br>c.−2C>G	**rs111604615 + rs201577108**<br>c.11G>C + c.16T>C	**rs71647871**<br>c.428G>A (p.G143E)	**rs2244613**<br>c.1171−33C>A
C	G - T	G	C
C	G - T	G	C
G	C - C	A	A
G	C - C	A	A

Table S3 Oligonucleotide probes immobilized on a biochip.

**rs12149368_C**	CCCTTCCA**C**GATGTGG–NH2	
**rs12149368_G**	CCCTTCCA**G**GATGTGG–NH2	
**rs111604615_G + rs201577108_T**	GTGGCTCC**G**TGCC**CT**T–NH2	*
**rs111604615_C + rs201577108_C**	GTGGCTCC**C**TGC**TC**T–NH2	*
**rs71647871_G**	CATCAGC**C**CCCCTCC–NH2	**
**rs71647871_A**	CCATCAGC**T**CCCCTCC–NH2	**
**rs2244613_C**	CTCACC**C**GGGGCTG–NH2	
**rs2244613_A**	GCTCACC**A**GGGGCTG–NH2	

* The Probe Sequences also includes ss1425684459 (c. 15C>T), that also is part of *CES1A1c*
** complementary strand probes

1.3. Biochip analysis.

1.2.1. Amplification of rs71647871 and rs2244613

One-step multiplex PCR was performed on genomic DNA as a template. The PCR mixture (25 μL) contained 1×PCR buffer (Syntol, Russia), 4 mM MgCl2, 0.4 mM of each dNTP (Sileks, Russia), 2.5 units of SynTaq DNA polymerase (Syntol), 1 pmol of each locus-specific primer (Suppl.Table 2), 100 pmol universal primer with Cy7 on 5'-end (5'-Cy7-TCATTGGATCTCATTA-3'), 8 μM of dUTP-Cy7, and 1 μL of DNA (5–50 ng/mL). The PCR program was as follows: 40 cycles of 94°C for 30 s, 62°C for 40 s, 64°C for 40 s, 66°C for 40 s, and 72°C for 20 s, followed by 40 cycles of 94°C for 30 s, 52°C for 10 s, and 72°C for 30 s.

Table S4. Primers for amplification of rs71647871 and rs2244613 for biochip analysis

**Primer name**	**Sequence (5′ – 3′)**
**rs71647871_F**	CAACAGGTAGGTCACCAAACAAGAC
**rs71647871_R**	tcattggatctcattaTCACCACCACCACGTTTTCATGG
**rs2244613_F**	TTCACCATTGAGGCAGGACTTT
**rs2244613_R**	tcattggatctcattaGAATCAGTTCCTTAGCAATGCACTG

1.2.2. Amplification of *CES1A1c* region (rs12149368, rs111604615 and rs201577108).

Nested PCR was used. At the first stage, a 2.6 kb region of the *CES1* gene from the promoter to intron 1 was amplified using LongAmp® Taq Hot Start (New England Biolab, UK) and primers

from Bjerre et.al., 2018 [33] (5'-ACTATGGGGGACGGAGxTxTxCxA-3' and 5'-CAGGAGC-TATTGAGAGATGGAAxTxCxAxT-3', “x” - phosphothioate bond).

The reaction was carried out in a volume of 15 μl, reaction composition 1× LongAmp Taq Reaction Buffer, 300 μM of each dNTP, 5 pmol of each primer, 2.5 units of LongAmp Hot Start Taq DNA Polymerase and 30 ng DNA. The PCR program was as follows: 10 cycles of 93°C for 15 s, 57°C for 30 s, 68°C for 3 min, followed by 20 cycles of 93°C for 15 s, 57°C for 30 s, 68°C for 4 min.

The obtained product was used as a template in the second stage of PCR with fluorescent labeling. The PCR mixture (25 μL) contained 1×PCR buffer (Syntol, Russia), 2 mM MgCl2, 0.4 mM of each dNTP (Sileks, Russia), 1.5 units of SynTaq DNA polymerase (Syntol), 5 pmol of forward primer (5'-GATCTCTCCCAATTAGAGGATTAGGC-3') and 50 pmol of reverse primer (5'-CCAA-GCCGCGGAAGCAG-3'), 8 μM of dUTP-Cy7, and 1 μL of DNA (PCR-product from previous step). The PCR program: 35 cycles of 94°C for 30 s, 62°C for 30 s, 72°C for 30 s.

1.2.3. Hybridization on a biochip and detection of results

The PCR product (10 μl from each reaction 1.2.1. and 1.2.2.) was placed into a biochip chamber with 10 μl formamide (Panreac, Spain) and 10 μl 20×SSPE buffer (Thermo Scientific). The biochips were incubated for 12 h at 37ºC and washed with 1×SSPE at room temperature for 10 min. The remaining buffer was washed off with water and the biochips were dried. Fluorescent signals were detected using a portable microarray analyzer and processed using ImageWare software (Biochip-IMB, Russia).

Figure S2. An example of analysis on a biochip

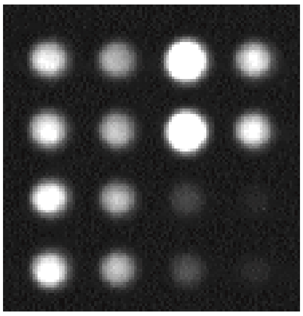

Genotype:
rs12149368_C/G
rs111604615_G/C
rs201577108_T/C
rs71647871_G/G
rs2244613_C/C

1.4. Sanger sequencing.

Sequencing of CES1A1c was performed on a 2.6 kb product (as described in **1.2.2)**, sequencing primer - 5'-CCAAGCCGCGGAAGCAG-3'.

For analysis of rs2244613, the product was amplified using primers 5'-AGTT-GCACAGGGCTTATGGGG-3' and 5'-GAATCAGTTCCTTAGCAATGCACTG-3', sequencing was performed using primer 5'-AGTTGCACAGGGCTTATGGGG-3'.

For analysis of rs71647871, the product was amplified from the promoter to intron 5 of the *CES1* gene, 12.5 kb in size. We used QIAGEN LongRange PCR Kit (Qiagen, Germany) and primers from Bjerre et.al., 2018 [33] (5'-ACTATGGGGGGACGGAGxTxTxCxA-3' and 5'- CCAG-TCCTGAATTCAGGTATTGTAATxCxA-3', “x” - phosphothioate bond). The reaction was carried out in a volume of 20 μL, reaction composition 1× PCR-Buffer, 500 μM of each dNTP, 10 pmol of each primer, 0.2 μL of LR-Enzyme mix and 30 ng DNA. The PCR program was as follows: 10 cycles of 93°C for 15 s, 60°C for 30 s, 68°C for 13 min, followed by 20 cycles of 93°C for 15 s, 60°C for 30 s, 68°C for 15 min.

Then this 12.5 kb PCR-product was used as a template in PCR with primers 5'-CAACAGGTAGGTCACCAAACAAGAC-3' and 5'-TCACCACCACCACGTTTTCATGG-3', and this product was sequenced from primer 5'-TCACCACCACCACGTTTTCATGG-3'.

PCR products were purified using the Cleanup Mini kit (Evrogen, Russia) or ethanol reprecipitation. DNA sequencing was performed using the ABI PRISM® BigDye™ Terminator v. 3.1 followed by analysis of the reaction products on an automatic sequencer Applied Biosystems 3730 DNA Analyzer.

2. Supplementary Results

Table S5. Baseline characteristics of patients between genotypes *CES1*.

rs2244613				
	A/A	A/C	C/C	p-value
**Gender**				0.967
fem	114	60	12	
male	62	31	7	
**Age, years (mean ± sd)**	67.88 ± 10.15	66.45 ± 10.5	65.32 ± 8.58	0.3706
**Weight, kg (mean ± sd)**	85.64 ± 18.65	82.41 ± 20	85.82 ± 17.59	0.3158
**BMI (mean ± sd)**	31.25 ± 6.04	30.03 ± 6.12	32.23 ± 7.18	0.3168
**GFR, ml/min (mean ± sd)**	69.02 ± 20	72.33 ± 19.99	76.61 ± 21.61	0.2052
**Single dose, mg (mean ± sd)**	7.2 ± 4.32	6.84 ± 4.61	6.71 ± 4.57	0.4092
CES1A1c				
	wt	wt/*CES1A1c*	*CES1A1c*/*CES1A1c*	
**Gender**				0.148
fem	124	56	6	
male	73	27	0	
**Age, years (mean ± sd)**	67.34 ± 10.54	66.84 ± 9.27	70 ± 10.97	0.8693
**Weight, kg (mean ± sd)**	84.6 ± 18.08	84.83 ± 21.51	83.38 ± 13.71	0.8185
**BMI (mean ± sd)**	30.94 ± 6.04	30.75 ± 6.62	32.6 ± 3.29	0.4644
**GFR, ml/min (mean ± sd)**	70.39 ± 20.35	70.02 ± 19.82	83.5 ± 15.81	0.2421
**Single dose, mg (mean ± sd)**	7.13 ± 4.38	6.72 ± 4.4	9.17 ± 6.06	0.3491
rs71647871				
	G/A	G/G	A/A	
**Gender**				1
fem	5	181	-	
male	2	98	-	
**Age, years (mean ± sd)**	69.71 ± 14.66	67.19 ± 10.06	-	0.4354
**Weight, kg (mean ± sd)**	88.21 ± 19	84.55 ± 19.04	-	0.5727
**BMI (mean ± sd)**	31.33 ± 5.56	30.91 ± 6.19	-	0.5532
**GFR, ml/min (mean ± sd)**	70 ± 29.96	70.58 ± 19.92	-	0.7656
**Single dose, mg (mean ± sd)**	6.79 ± 2.38	7.06 ± 4.46	-	0.6506

GFR – glomerular filtration rate, BMI – body mass index, sd – standard deviation

Table S6. Peak and trough enalaprilat concentrations based on *CES1* genotypes in patients receiving different doses of enalapril.

		**Peak enalaprilat concentration**				**Trough enalaprilat concentration**			
**Single dose, ng**	**n**	**1st_Qu**	**Median**	**Mean**	**3rd_Qu**	**1st_Qu**	**Median**	**Mean**	**3rd_Qu**
CES1 rs2244613									
AA									
2.5	28	11.02	20.16	28.7	34.27	7.82	11.79	16.21	18.12
5	76	17.46	31.14	33.76	40.48	7.945	14.03	18.006	25.195
7.5	10	24.64	32.44	37.84	48.76	14.52	15.63	21.1	22.54
10	47	37.85	55.75	59.18	69.55	14.71	22.31	25.79	33.27
12.5	1	106.6	-//-//-	-//-//-	-//-//-	57.78	-//-//-	-//-//-	-//-//-
15	4	26.46	48.13	68.86	90.54	19.03	29.22	29.26	39.45
20	10	43.76	75.08	98.27	133.34	35.56	42.08	74.28	94.11
AC									
2.5	23	7.615	10.07	14.471	18.78	3.69	5.75	6.73	10.58
5	36	17.26	27.34	33.83	46.53	9.658	17.01	20.826	27.297
7.5	3	29.06	30.34	38.01	43.12	10.9	12	12.98	14.56
10	19	19.52	40.06	41.31	53.98	12.83	20.9	20.59	28.05
12.5	1	86.61	-//-//-	-//-//-	-//-//-	32.07	-//-//-	-//-//-	-//-//-
15	4	59.84	62.45	64.77	67.38	16.79	25.32	22.51	31.05
20	5	44.29	56.08	84.12	123.14	25.01	28.35	33.72	46.41
CC									
2.5	4	6.048	9.55	12.12	15.623	6.625	9.8	10.713	13.887
5	9	12.79	18.08	22.52	28.75	3.53	8.03	9.696	11.59
7.5	1	26.21	-//-//-	-//-//-	-//-//-	15	-//-//-	-//-//-	-//-//-
10	3	27.17	38.98	33.95	43.24	9.05	11.55	10.31	12.2
12.5	0	-	-	-	-	-	-	-	-
15	1	27.08	-//-//-	-//-//-	-//-//-	11.8	-//-//-	-//-//-	-//-//-
20	1	104.4	-//-//-	-//-//-	-//-//-	42.09	-//-//-	-//-//-	-//-//-
CES1A1c									
WT									
2.5	34	9.467	17.505	23.739	26.65	4.785	10.58	13.309	15.005
5	86	16.67	26.41	32.22	41.34	8.4	14.9	18.42	25.37
7.5	9	27.78	30.56	41.63	55.9	15	17.13	22.3	23.45
10	50	34.49	46.92	54.4	64.89	14.27	21.65	23.88	30.35
12.5	1	86.61	-//-//-	-//-//-	-//-//-	32.07	-//-//-	-//-//-	-//-//-
15	6	27.52	40.8	58.43	60.21	10.27	16.96	21.1	27.81
20	11	51.13	104.38	103.37	130.87	36.86	45.18	72.53	84.61
het (wt/*CES1A1c*)									
2.5	20	9.81	13.29	18.44	21.98	5.49	7.885	9.945	12.768
5	34	22.88	33.13	35.13	42.48	8.758	14.475	18.11	25.883
7.5	4	28.48	32.33	33.01	36.85	11.05	11.84	12.31	13.1

10	17	36.18	57.26	52.93	65.04	11.65	22.31	24.4	36.4
12.5	1	106.6	-//-//-	-//-//-	-//-//-	57.78	-//-//-	-//-//-	-//-//-
15	3	64.81	67.42	70.35	74.42	27.99	35.05	30.77	35.69
20	4	42.67	50.19	52.84	60.35	25.73	33.17	32.66	40.09
mut (*CES1A1c/CES1A1c*)									
2.5	1	8.91	-//-//-	-//-//-	-//-//-	0	-//-//-	-//-//-	-//-//-
5	1	20.28	-//-//-	-//-//-	-//-//-	6.31	-//-//-	-//-//-	-//-//-
7.5	1	11.96	-//-//-	-//-//-	-//-//-	15	-//-//-	-//-//-	-//-//-
10	2	19.75	24.14	24.14	28.54	12.1	12.64	12.64	13.19
12.5	0	-	-	-	-	-	-	-	-
15	0	-	-	-	-	-	-	-	-
20	1	159.3	-//-//-	-//-//-	-//-//-	25.01	-//-//-	-//-//-	-//-//-
CES1 rs71647871									
GG									
2.5	55	9.375	14.63	21.542	25.805	4.75	8.9	11.84	14.12
5	117	17.08	28.3	32.64	41.34	8.037	13.935	17.627	24.867
7.5	13	26.21	30.34	37.26	50.2	14.36	15.48	19.44	19.79
10	67	34.09	47.56	53.62	65.15	13.3	22.26	24.16	31.16
12.5	2	91.61	96.61	96.61	101.62	38.5	44.92	44.92	51.35
15	9	28.85	62.21	62.4	67.42	11.8	22.12	24.32	35.05
20	16	42.67	75.08	94.23	127	31.27	40.53	59.59	54.64
GA									
2.5	0	-	-	-	-	-	-	-	-
5	4	26.37	33.27	41.7	48.59	21.09	30.32	35.69	44.91
7.5	1	34.32	-//-//-	-//-//-	-//-//-	12.2	-//-//-	-//-//-	-//-//-
10	2	32.55	37.72	37.72	42.9	4.838	7.835	7.835	10.832
12.5	0	-	-	-	-	-	-	-	-
15	0	-	-	-	-	-	-	-	-
20	0	-	-	-	-	-	-	-	-

1st_Qu – first quartile, 3rd_Qu – third quartile. In groups where n = 1, the only value is shown in the "1st_Qu" column, in the remaining columns the sign '-//-//-' is indicated.

Table S7. Distribution of patients according to correspondence of the therapeutic range of enalaprilat, depending on the *CES1* rs2244613 genotype

	**AA**	**AC**	**CC**
**Peak enalaprilat concentration**			
>50	55	22	1
10 – 50	112	56	15
<10	9	13	3
**Trough enalaprilat concentration**			

>50	15	2	0
10 – 50	117	58	10
<10	43	31	9
